# Switching Off Vascular MAPK Signaling: A Novel Strategy to Prevent Delayed Cerebral Ischemia Following Subarachnoid Hemorrhage

**DOI:** 10.1007/s12975-024-01234-z

**Published:** 2024-02-09

**Authors:** Lars Edvinsson, Diana N. Krause

**Affiliations:** 1 Division of Experimental Vascular Research, Department of Clinical Sciences, Lund University, Sölvegatan 19, 22100 Lund, Sweden; 2 Department of Experimental Research, Glostrup Research Institute, CopenhagenUniversity, Copenhagen, Denmark; 3 Department of Pharmaceutical Sciences, SchoolofPharmacy&PharmaceuticalSciences, University of California at Irvine, Irvine, CA USA

**Keywords:** Subarachnoid hemorrhage, Cerebral vasculature, MAPK kinase inhibitor, Delayed cerebral ischemia, Vasoconstriction

## Abstract

Patients who initially survive the rupture and repair of a brain aneurysm often take a devastating turn for the worse some days later and die or suffer permanent neurologic deficits. This catastrophic sequela is attributed to a delayed phase of global cerebral ischemia (DCI) following aneurysmal subarachnoid hemorrhage (aSAH), but we lack effective treatment. Here we present our view, based on 20 years of research, that the initial drop in blood flow at the time of rupture triggers genomic responses throughout the brain vasculature that manifest days later as increased vasoconstriction and decreased cerebral blood flow. We propose a novel treatment strategy to prevent DCI by early inhibition of the vascular mitogen-activated protein kinase (MAPK) pathway that triggers expression of vasoconstrictor and inflammatory mediators. We summarize evidence from experimental SAH models showing early treatment with MAPK inhibitors “switches off” these detrimental responses, maintains flow, and improves neurological outcome. This promising therapy is currently being evaluated in clinical trials.

## Introduction

Aneurysmal subarachnoid hemorrhage (aSAH) is the cause of a devastating type of stroke (5%) that often strikes in midlife and results in a high degree of fatality and morbidity [1]. The sudden rupture of an aneurysm in the wall of a cerebral artery leads to bleeding into the subarachnoid space. In patients who survive the initial insult, the aneurysm is surgically repaired to stop the bleeding, either by clipping or intravascular coiling. Yet several days later, many of the patients (> 30%) take a dramatic turn for the worse resulting in death or serious long-term disability [2]. The cause is attributed to delayed cerebral ischemia (DCI) but currently there is no effective treatment to prevent this sequela and improve outcome [1–4].

Considerable effort has gone into trying to understand the complex pathophysiology underlying DCI. The clinical time course is well-defined; symptoms appear 2–4 days post aSAH, peak at 5–7 days, and last up to 14 days [2, 5, 6]. Neurological deterioration is associated with decreased cerebral blood flow (CBF), global brain ischemia, inflammation, blood–brain barrier (BBB) disruption, cerebral edema, and brain injury [2, 4, 5]. For many years, DCI was thought to result from the dramatic vasospasm of large cerebral arteries that is seen angiographically at 3–5 days after aSAH [7]. However, drugs aimed at alleviating this delayed vasospasm have failed to improve outcome in SAH [5, 6]. A disappointing recent example is the endothelin receptor antagonist clazosentan [8]. Despite the fact it relaxed large artery vasospasm and countered the impact of elevated release of the potent vasoconstrictor endothelin-1 (ET-1), this drug was unsuccessful in clinical trials of aSAH [6, 9, 10].

The current view of DCI now recognizes a more complex underlying pathology that notably encompasses the whole brain and not just the site of rupture [2, 4–6]. Key changes during the subacute phase after aSAH include microcirculatory constriction, release of inflammatory mediators such as cytokines and chemokines, microthrombosis, and activation of matrix metalloproteinases (MMPs) and apoptotic pathways [2, 4, 6, 11]. This multifaceted pathophysiology poses a treatment challenge as it is unlikely any therapy aimed to alleviate just one of these changes will prevent DCI.

## Novel Treatment Strategy

**Fig. 1:**
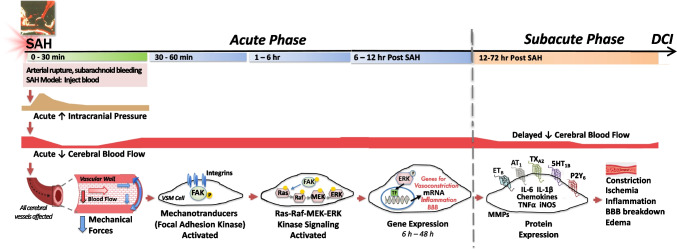
Vascular changes in response to SAH. Schematic illustration of the time course of events following SAH with particular emphasis on the evolving response of vascular smooth muscle in cerebral arteries and microvessels as described in the text. Abbreviations: SAH subarachnoid hemorrhage, VSM vascular smooth muscle, FAK focal adhesion kinase, MEK mitogen-activated protein kinase kinase, ERK1/2 extracellular signal-related kinase, TF transcription factor, MMP matrix metalloproteinase, BBB blood–brain barrier, DCI delayed cerebral ischemia

**Fig. 2:**
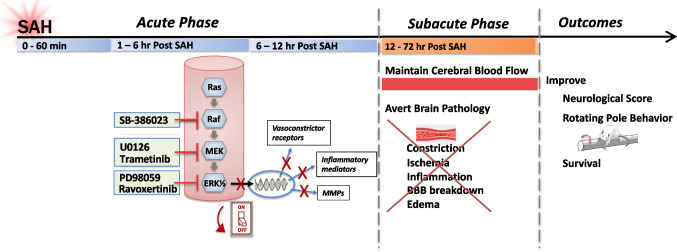
Effect of raf, MEK1/2, and ERK1/2 inhibitors in experimental SAH. Rationale for the proposed novel therapy to prevent DCI following SAH based on studies in experimental animal models

We have developed a novel strategy, reviewed here, that shows promising results in preclinical SAH models and is currently being evaluated in a phase II clinical trial. Our approach is designed to switch off initiation of the injury cascade at an early stage to prevent expression of phenotypic changes that underlie DCI. The focus is on the cerebral vasculature as the central player in SAH pathology. Our research has identified key changes in smooth muscle cells of cerebral arteries and microvessels that occur in response to SAH [12]. We found that the intracellular mitogen-activated protein kinase (MAPK) signaling pathway is activated within 6 h of the hemorrhagic insult, and this initiates an evolving, comprehensive genomic program of injury responses including upregulation of multiple vasoconstrictor receptors and inflammatory mediators (Fig. 1) [13–15]. Our most significant, and somewhat surprising, discovery is that when specific inhibitors are given early to block the vascular MAPK pathway, one can prevent delayed cerebral ischemia and injury and improve neurological outcome and survival in experimental SAH models (Fig. 2) [16–19].

## Vascular Changes After SAH

A key trigger for DCI occurs at the time of aneurysm rupture when a sudden, sharp increase in intracranial pressure and decrease in cerebral blood flow abruptly reduce the mechanical forces exerted on brain blood vessels (Fig. 1) [13, 17, 20, 21]. Normally, cells of the vessel wall are continuously exposed to dynamic mechanical forces—fluid shear stress, hydrostatic pressure, and cyclic stretch—that are critical cues for maintaining healthy vascular function [22]. Endothelial and vascular smooth muscle cells use specialized mechanosensors, such as integrins, Piezo channels, and membrane receptors, to detect and transduce mechanical changes into adaptive vascular responses that maintain blood flow [22–24].

As first described by Bayliss [25], vascular myocytes initially react with an autoregulatory myogenic response, such as vasodilation to counteract a decrease in transmural pressure. In SAH, however, acute measures are not able to restore blood flow, so additional mechanisms for long-term adaptation are triggered via intracellular signaling and altered gene expression (Fig. 1). It is important to note that in SAH, blood vessels throughout the brain are impacted by loss of flow including microvessels in brain regions far downstream from the site of aneurysm rupture [4, 26–28]. The acute elevation in intracranial pressure (ICP) and global hypoperfusion that occurs after aneurysm rupture is thought to initiate the subsequent and widespread microvascular dysfunction that develops during DCI [13, 27, 29].

## Upregulation of Vasoconstriction

Using a well-characterized rat model of aSAH [30, 31], we have identified a number of phenotypic changes that occur in cerebrovascular smooth muscle cells several days after SAH induction [12]. Of particular relevance to DCI, cerebral arteries develop greater sensitivity to vasoconstrictors due to increased expression of specific smooth muscle receptors. Our initial study in 2003 demonstrated that, 2 days after experimental SAH, there was a selective increase in expression and function of endothelin ET_B_ receptors in vascular smooth muscle that mediate vasoconstriction [32]. A similar change in vascular ET_B_ receptors was reported after SAH in monkeys [33]. In subsequent studies, we found that additional vasoconstrictor receptors were upregulated in cerebrovascular smooth muscle after SAH, i.e., angiotensin type 1 receptors (AT_1_) [34], 5-hydroxytryptamine type 1B receptors (5-HT_1B_), thromboxane TX_2A_ receptors [35], and recently, P2Y_6_ purinergic receptors [36]. In large cerebral arteries, the increase in multiple receptors that mediate vasoconstriction is likely a key factor underlying the classic vasospasm seen angiographically after aSAH [37].

In studies of contractile ET_B_, AT_1_, 5-HT_1B_, TX_2A_, and P2Y_6_ receptors, we demonstrated SAH induces expression of receptor mRNA [32, 35, 38] and protein that is localized in the smooth muscle layer of cerebral arteries as well as intracerebral microvessels [17, 18, 34–36]. Using in vitro contractile assays to assess function, we showed that stimulating these receptors produces greater constrictor responses in arteries taken from SAH animals [32, 35, 39]. In addition, contractile responses to potassium-induced depolarization are increased [40], which is further evidence that a general vasoconstrictive phenotype develops in cerebral arteries after SAH. Intraparenchymal arterioles also show a persistent constricted morphology that is readily apparent in resin casts of the cerebral microvasculature taken at 3 and 5 days following experimental SAH [26]. In vivo imaging in mice found that more than 70% of cerebral arterioles examined were constricted at 3 days after experimental SAH, with the smaller arterioles exhibiting the greatest constriction [28].

## Correlation with Decreased CBF

It is now recognized that insufficient blood flow is a primary cause of delayed brain injury following SAH [27, 41–43]. Decreased global CBF during the subacute phase after SAH is correlated with the occurrence of DCI and poor patient outcomes [41, 42]. In experimental SAH, the time course for increasing vasoconstrictor receptors and vascular reactivity also correlates with a secondary reduction in global and regional cerebral blood flow that occurs 12–72 h after experimental SAH (Fig. 1) [13, 17, 18, 21, 26, 35, 39].

In our experimental SAH studies, vascular receptor mRNA expression starts to increase around 6 h post-SAH with a peak at 12–24 h [39]. Receptor protein expression then increases with a maximum effect at 24–48 h after SAH. Corresponding vasoconstrictor responses intensify in parallel with protein expression. The receptor changes show a clear temporal correlation with a progressive decrease in regional CBF over the 24–48-h period post-SAH (Fig. 1) [39]. Moreover, the duration of the acute drop in CBF at the time of aneurysm rupture influences the extent of delayed upregulation of contractile receptors and correlates with the severity of CBF reduction measured at 3 days post-SAH [13].

Regarding the time course of events following a hemorrhagic insult, it should be noted that several terms are used. “Early brain injury” (EBI) refers to the pathophysiological changes occurring in the first 72 h [4, 42]. This time period is also called the subacute phase [5, 31] and involves disease mechanisms leading to the subsequent development of DCI in the delayed or chronic phase lasting 3–14 days post-SAH [5]. It should also be noted that rodent models of SAH show a similar progression of events as in human patients, however, specific pathological events may occur with a faster timeline [4].

## Upregulation of Vascular Inflammation and Remodeling

aSAH induces delayed inflammatory responses throughout the brain that contribute to cerebrovascular dysfunction, EBI, and poor outcome after SAH [4, 44–46]. The initial drop in blood flow causes global ischemia and triggers early expression of pro-inflammatory mediators in the vascular wall [47]. In our experimental SAH model, expression of cytokines and chemokines is induced in both cerebral arteries and microvessels [14, 38, 48, 49]. Microarray analysis revealed that inflammatory genes (interleukins IL-6 and IL-1beta, necrosis factor alpha (TNF-alpha), inducible nitric oxide synthase (iNOS), and chemokine ligands (CCL20, CXCL1, and CXCL2)) are the largest group of vascular genes differentially expressed after SAH [38]. The pro-inflammatory genes show an initial spike in mRNA expression during the first 3–6 h after experimental SAH followed by a delayed increase of longer duration that starts 12–24 h post-SAH and remains elevated at 48 h (Fig. 1) [38]. Protein expression of inflammatory mediators in the vasculature, which increase over a period of 1–3 days post-SAH, is found in the walls of both cerebral arteries and microvessels (Fig. 1) [14, 48, 49].

A study of brain samples taken after experimental SAH also found early mRNA expression of pro-inflammatory genes (2–8 h) [50]. This effect occurred throughout the brain, not just near the site of insult, which reflects the global decrease in blood flow and early period of widespread ischemia. During the subacute phase (24–48 h post-SAH), a robust increase in pro-inflammatory markers has been found throughout the brain [4, 46, 51, 52]. This period corresponds to that of the secondary decrease in regional CBF. The vasculature is an active participant in the inflammatory response, but at this point, other cell types are also involved including neurons, glia, microglia, and infiltrating immune cells such as macrophages and neutrophils [4, 53, 54].

Extracellular matrix–regulating genes, such as matrix metalloproteinases MMP8, MMP9, and MMP13, constitute another key group of genes that are transcribed in the vessel wall in the early post-SAH period (Fig. 1) [38]. In our experimental SAH model, vascular levels of mRNA for MMP8 and MMP9 peak at 3 h but remain elevated at 48 h post-SAH. Protein expression of these enzymes, which was localized in the cerebrovascular smooth muscle, increases over a period of 1–3 days post-SAH (Fig. 1) [14, 49]. Matrix metalloproteinases are involved in BBB breakdown [14, 38, 48, 49]. The early loss of BBB integrity after SAH is a major cause of the edema and inflammatory responses that contribute to early brain injury [55].

## SAH Activation of Vascular Intracellular Signaling

Our aim has been to identify early signaling pathways that are activated within the vasculature after SAH as these initial steps provide strategic targets for preventing the evolving pathology underlying DCI. A seminal discovery was that MAPK signaling is activated in brain arteries and intracerebral microvessels in response to experimental SAH [14, 15, 18, 49, 56, 57]. The role of the MAPK pathway in cells is to transduce, amplify, and integrate extracellular signals from the cell surface to the nucleus to alter gene expression and induce various cellular responses such as inflammation and differentiation [58–60]. The pathway consists of a cascade of intracellular protein serine/threonine kinases, ras → raf → MEK1/2 (mitogen-activated protein kinase kinase) → ERK1/2 (extracellular signal-related kinase, also called MAPK), in which each enzyme switches on the next through phosphorylation.

There is a rapid and dramatic increase in the phosphorylated, active form of ERK1/2 (pERK1/2) in both larger cerebral arteries and microvessels in response to experimental SAH [14, 15, 48, 49, 57]. Levels of pERK1/2 are elevated as early as 1 h post-SAH, remain high for 2–3 days, and then drop by day 4 (Fig. 1) [14, 15, 48, 49, 57]. The extent to which ERK1/2 is phosphorylated depends on the duration of the acute drop in CBF after SAH [13]. Early ERK1/2 activation after SAH is cell-specific as pERK1/2 immunohistochemistry could be visualized in the vascular smooth muscle but not the surrounding neuropil, nor could it be detected in cerebral vessels from control animals [18]. Interestingly, while vascular ERK1/2 is activated during the first 24 h after SAH, other kinase signaling pathways, i.e., p38 and JNK 2, are not turned on until 48 h later [57].

Stimulation of ras-raf-MEK1/2-ERK1/2 signaling is likely a consequence of early activation of vascular focal adhesion proteins involved in mechanotransduction (Fig. 1). Phosphorylation of focal adhesion proteins, including focal adhesion kinase (FAK), zyxin, and tensin-1, is increased in cerebral arteries within the first hour after experimental SAH (Fig. 1) [15]. This activation signals a change in mechanical forces on the vascular wall as a consequence of decreased CBF [15, 22]. FAK, in particular, is a known activator of the MAPK pathway [61] and thus it appears to be a key initiator of the vascular responses exhibited after SAH. We validated this concept by comparing cerebral arteries that were either mechanically stretched or unstretched in vitro for 12 h. Unstretched arteries exhibited an increase in ET_B_-mediated contraction and this effect could be blocked by inhibitors of either FAK or the MEK-ERK1/2 pathway [62].

Once activated, pERK1/2 goes on to phosphorylate a wide variety of cytosolic proteins, regulatory molecules, and nuclear transcription factors [58, 60]. At this point, the vascular response to SAH becomes greatly amplified with the induction of multiple pathological processes that contribute to early brain injury and DCI. For example, we showed that downstream pERK1/2 targets, transcription factors STAT3, ATF-2, and Elk-1, are rapidly phosphorylated in the arteries within the first 6 h after SAH [15, 48, 63]. These phosphorylated factors initiate gene expression by binding to a variety of gene promoters, including those for inflammatory and MMP proteins [15, 48] that then appear in the vessels 1–3 days later (Fig. 1) [14, 48, 49].

## Effect of MAPK Inhibitors on Vascular Changes

To confirm that the MAPK pathway plays a key role in initiation of SAH-induced vascular changes, we tested specific inhibitors of either raf (SB386023-b) [14, 17, 18, 57] or MEK1/2 (U0126, trametinib) [13, 16, 19, 49, 64] in the rat model [49]. Activation of pERK1/2 in cerebral arteries and microvessels is prevented if raf or MEK inhibitors are administered intracisternally within 6 h after SAH [18]. Moreover, this treatment blocks the upregulation of vasoconstrictor receptors [14, 16–18, 57] and expression of vascular inflammatory mediators and MMPs [14, 49] that occurs 2–3 days after SAH. Most importantly, global and regional cerebral blood flow is preserved in the subacute phase after early treatment with raf and MEK1/2 blockers [14, 17, 18]. We have validated that inhibition of the MAPK pathway has similar effects in male and female animals subjected to SAH [19]. Together these results demonstrate the significant impact of switching off the vascular MAPK pathway early on to prevent subsequent vascular pathology and maintain cerebral blood flow in the subacute phase.

## Organ Culture Model

We have characterized a useful in vitro model that mimics the vascular changes after SAH [12]. Isolated arteries placed in organ culture, where they are devoid of mechanical stresses for 1–2 days, show similar phenotypic changes, e.g., activation of focal adhesion kinase and pERK1/2 [62, 65, 66] as well as upregulation of vasoconstrictor receptors and inflammatory mediators [65–69]. Using this model, we confirmed that human cerebral arteries exhibit similar responses as those in rat arteries after experimental SAH [70, 71]. The constrictor and inflammatory changes that occur in vitro are prevented by inhibitors of Raf, MEK1/2, or ERK1/2 [66, 67, 70, 72]. Thus, this model provides a convenient screening tool for evaluating potential drugs for SAH [66, 73].

## RAF/MEK/ERK Inhibitors Improve SAH Outcome

Ultimately, the goal is to identify a treatment that will deliver better outcomes for SAH patients. To this aim, inhibitors of the MAP kinase pathway were evaluated for their ability to improve neurological function after experimental SAH in vivo [13, 16, 18, 19, 64, 73]. Animals exhibit reduced CBF and neurological deficits 2–3 days after SAH. They have difficulties traversing a rotating horizontal pole, which is a test of balance and gross sensorimotor function as well as memory and motivation. Behavioral assessments indicate lower neurologic scores and diminished well-being. However, administration of a raf (SB386023-b) or MEK1/2 inhibitor (U0126, trametinib) within 6 h post-SAH prevented decreased CBF and neurological deficits from occurring 2–3 days later in both males and females [13, 16, 18, 19, 64, 73]. Survival in rats also was increased by early treatment with U0126 [13]. Similarly, it has been shown in a collagenase mouse model of intracerebral hemorrhage that U0126, injected ICV at 2 h post-stoke, improves functional recovery assessed 1–2 days later [74]. Several other groups have reported that administration of specific ERK1/2 inhibitors (ravoxertinib, PD98059) within 1 h of experimental SAH will also alleviate long-term neurobehavioral deficits [75, 76].

## The Importance of Timing

One of the challenging aspects of understanding and developing treatment for delayed pathology after SAH is that underlying mechanisms evolve over time. Early SAH-induced signals are potential therapeutic targets, but only if they are activated within a clinically relevant time window for administration of pharmacological inhibitors. For example, we considered the potential of a FAK inhibitor which prevented ERK phosphorylation and ET_B_ expression in unstretched arteries in vitro when added at time 0 [62]. However, SAH activation of FAK is a very early, transient event that is seen at 1 h, but not 6 h, after the SAH insult [15]. Thus, FAK inhibitors would be of limited use in the clinic. On the other hand, the appropriate time window for targeting the MAPK pathway is very favorable. If the treatment is started within the first 6 h after experimental SAH, raf and MEK inhibitors effectively prevent subsequent vascular changes and the decrease in CBF in the subacute period [14–18]. Interestingly, these inhibitors are not effective if treatment is started 12 h after SAH [14, 18] suggesting the “amplification switch” has already been turned on by this time and downstream processes are well under way.

Another consideration is how long to treat with a MAPK inhibitor. Vascular pERK1/2 remains elevated for several days after experimental SAH [14, 15, 48, 49, 57], suggesting treatment with an inhibitor may be warranted over this time period. Indeed, a comparison of different treatment regimens in the rat model showed that three doses of U0126, administered at 6, 12, and 24 h post-SAH, resulted in better outcomes as compared to a single dose given at 6 h [77]. Treatments of longer duration, however, could paradoxically worsen recovery as activation of the MAPK pathway in later phases contributes to vascular and neuronal recovery after ischemia [60, 78]. Further study is needed to clarify the time course during which initial deleterious responses to ERK signaling after SAH may transition to beneficial repair mechanisms.

## Targeting the “Switch” vs. Selected Changes in SAH

It is our view that selectively targeting a specific factor will not be sufficient to alleviate delayed cerebral ischemia and improve clinical outcome after SAH. The risk of DCI appears to be determined by numerous pathological changes that evolve over time [4–6]. Thus, targeting an early biological “switch” that triggers multiple downstream changes, such as inhibition of the ras-raf-MEK1/2-ERK1/2 pathway, is proposed as a better treatment strategy for preventing DCI (Fig. 2). This is illustrated by a direct comparison of a MEK1/2 inhibitor, U0126, with the endothelin ET receptor antagonist clazosentan in the rat SAH model [8]. The rationale for clazosentan treatment was that it would block endothelin-mediated vasospasm and thereby prevent DCI [5, 6]. In experimental SAH animals, clazosentan did inhibit contractile responses to endothelin; however, it did not affect neurologic outcome [8]. This finding mirrors the disappointing results from failed clinical trials of clazosentan [5, 29]. In contrast, U0126 had no vasomotor effect by itself in the SAH model but it prevented upregulation of numerous constrictor receptors, including ET_B_, in cerebral arteries and significantly improved neurologic outcome [8]. Currently, there are a number of pharmacological agents being evaluated for prevention of DCI in aSAH [29]. However, these potential treatments, like clazosentan, primarily focus on just one target involved in post-SAH pathology, e.g., interleukin-1 receptor antagonist, PDE-III inhibitor, NMDA antagonist, and direct vasodilators [29].

For over 30 years, oral nimodipine has been the only drug specifically recommended for treating aSAH [5, 29]. Nimodipine is a dihydropyridine blocker of L-type voltage-gated Ca^2+^ channels and it was originally developed as a means to prevent the vasospasms observed angiographically after aSAH [7]. While clinical studies of nimodipine show modest efficacy in improving aSAH outcome, its mechanism of action, as well as the role of angiographic vasospasm, has come into question [29].

Using our rat SAH model, we directly compared behavioral outcome and vascular effects of nimodipine and the MEK1/2 inhibitor U0126 [77]. U0126 has been formulated for intrathecal administration so nimodipine was also tested intrathecally as well as subcutaneously. Both U0126 and nimodipine improved rotating pole behavior at 48 h post-SAH; however, the latter drug was only effective when administered subcutaneously [77]. Clinically, only oral, but not intraventricular, administration of nimodipine has shown clear benefit in aSAH [29]. However, this route of administration is not ideal. There is significant risk of systemic hypotension that can necessitate dose reduction or drug discontinuation that limits drug efficacy [29, 79]. In contrast, intrathecal U0126 has no effect on mean arterial pressure [77]. A promising new approach for nimodipine treatment is the use of an extended-release microparticle formulation that can be administered intraventricularly or intracisternally to increase drug levels in the brain and avoid systemic toxicity [80].

Beneficial effects of nimodipine in aSAH were initially attributed to its ability to vasodilate cerebral arteries and thereby prevent delayed vasospasm [7]. However, the current view is that large artery vasospasm is not a primary cause of DCI, and nimodipine may act not just on the vasculature but through other mechanisms to improve aSAH outcome [29]. In our rat SAH model, we looked specifically at cerebrovascular effects of nimodipine in comparison to U01026. As expected, U0126 inhibited upregulation of both ET_B_ receptor expression and endothelin-mediated vasoconstriction; however, nimodipine treatment had no effect on vascular receptor expression but actually increased constriction via non-L-type, voltage-insensitive Ca^2+^ channels [77]. These findings point to possible negative effects of nimodipine on vascular reactivity.

## MEK Inhibitor in Clinical Trials of aSAH

Early inhibition of MEK1/2-ERK1/2 signaling has the potential to improve outcome after aSAH. Moreover, this novel therapeutic strategy can be implemented within a clinically relevant time window. To evaluate this treatment strategy, we have initiated clinical testing of a MEK1/2 inhibitor in aSAH patients (EudraCT number 2013–003690-10). U0126 (1,4-diamino-2,3-dicyano-1,4-bis (2-aminophenylthio) butadiene) was formulated with cremophor for intrathecal administration in the study [19, 81]. A phase Ib trial of U0126 in patients with aSAH has been completed with no serious drug-related side effects. A phase II randomized controlled trial for severe aSAH (STOP-DCI) is now underway in which U0126 is given intraventricularly at three timepoints (6–8, 12, and 24 h post-SAH). The primary outcome measurement is taken 4 weeks later using the Glasgow Coma Scale-Extended. Completion of the trial is expected in 2024.

## Conclusion

We have summarized here the rationale and experimental foundation for a novel treatment strategy to prevent delayed ischemia (DCI) that occurs after aSAH with devastating consequences. Based on over 20 years of research, we propose that the acute drop in cerebral blood flow following aneurysm rupture initiates a genomic response in smooth muscle cells throughout the brain vasculature that then manifests days later as increased vasoconstriction, decreased cerebral blood flow, and subsequent brain ischemia (Fig. 1). We have identified a key intracellular signaling “switch,” the MAPK pathway, that is activated in the vasculature within 6 h after experimental SAH and is responsible for stimulating gene expression for vasoconstrictor receptors as well as inflammatory mediators. We propose that inhibitors of raf, MEK1/2, or ERK1/2 kinases will improve outcome when administered to aSAH patients within the first 6 h after aneurysm rupture. Indeed, these inhibitors have been shown in rodent SAH models to prevent upregulation of vasoconstrictor receptors and inflammatory mediators, maintain cerebral blood flow in the subacute phase, and improve neurologic outcome and survival (Fig. 2).

There are two important concepts that distinguish the MAPK inhibition strategy from other approaches proposed to alleviate DCI in aSAH. The first is the specific focus on cerebral blood vessels throughout the brain with the goal to prevent the delayed global drop in blood flow that causes a secondary period of brain ischemia. We hypothesize that the acute reduction in flow and mechanical forces within the blood vessels at the time of aSAH is the trigger that initiates subsequent adaptive/maladaptive changes in the smooth muscle to enhance vasoconstriction. Although many other cell types, e.g., neurons, glia, and various immune cells, become involved in the pathology that emerges during DCI [4, 6], we posit that their responses are a reaction to the delayed ischemic insult. If flow is not first preserved, the effectiveness of drugs for neuroprotection, antiinflammation, etc., will be limited.

Interest in the role of vasoconstriction after aSAH is not new, but initially the focus was on angiographically observed, delayed vasospasm in large cerebral arteries adjacent to the aneurysm rupture [2, 6, 7]. It is now becoming apparent that vasoconstriction occurs throughout the brain, however, the proposed treatments to alleviate this have focused on a single mechanism, e.g., endothelin receptors [6, 11, 29]. This approach has not been sufficient to improve outcome. Thus, the second unique aspect of our proposed strategy is that the goal is to suppress as many pathological endpoints as possible by inhibiting the initial reaction of the vessels to low flow. What is remarkable about our findings is that we have discovered at least one key “switch,” the MAPK pathway, that is responsible for turning on multiple genes involved in vasoconstriction, inflammation, and BBB remodeling. This is likely not the only injury pathway triggered, but it appears to have sufficient impact that switching it off can prevent delayed injury after SAH.

## Data Availability

The data presented in this journal have been published and approved in peer reviewed international journals.
